# *Candida auris:* What Have We Learned About Its Mechanisms of Pathogenicity?

**DOI:** 10.3389/fmicb.2018.03081

**Published:** 2018-12-12

**Authors:** Luana Rossato, Arnaldo Lopes Colombo

**Affiliations:** Special Mycology Laboratory, Universidade Federal de São Paulo, São Paulo, Brazil

**Keywords:** *Candida auris*, pathogenicity, virulence, genomics, phenotypic traits, thermic and osmotic stress, biofilms

## Abstract

*Candida auris* has emerged globally as a multidrug-resistant (MDR) medical care-associated fungal pathogen. Recent reports have demonstrated that *C. auris* usually expresses fewer virulence factors than does *Candida albicans*. However, the tendency of *C. auris* transmission within and between healthcare facilities is unique among *Candida* spp. and is possibly promoted by virulence and pathogenicity factors that facilitate skin colonization and environmental persistence. To understand the ability of this yeast to cause disease, we herein discuss several virulence and pathogenicity aspects of *C. auris.*

## Introduction

*Candida auris* is an emerging multidrug-resistant (MDR) yeast pathogen that causes healthcare-associated invasive infections. There is growing evidence suggesting that *C. auris* may persistently colonize the hospital environment and multiple body sites of patients, leading to high transmissibility, and prolonged outbreaks (Schelenz et al., 2016; Vallabhaneni et al., 2017). Indeed, we are encountering for the first time an MDR yeast pathogen with a high capacity to cause clusters of invasive infections in medical centers around the world (Kim et al., 2009; Lee et al., 2011; Chowdhary et al., 2013, 2014; Calvo et al., 2016; Prakash et al., 2016; Lockhart et al., 2017; Morales-Lopez et al., 2017). *C. auris* fungemia results in crude mortality rates ranging from 32 to 66%, depending on the patient’s underlying conditions, geographic region, and age as well as the clinical management of the infection (Lee et al., 2011; Sarma et al., 2013; Chowdhary et al., 2014; Calvo et al., 2016; Schelenz et al., 2016; Morales-Lopez et al., 2017; Vallabhaneni et al., 2017).The description of *C. auris* fungemia outbreaks worldwide illustrates the dramatically increased need to develop new strategies to prevent and control infections due to this multiresistant pathogen. Despite the large number of papers addressing *C. auris* infections published since 2009, few have attempted to investigate the pathogenicity and virulence of this new human pathogen. Here, we review all papers addressing biological factors that affect the pathogenicity of *C. auris*.

## What We Have Learned From Comparative Genomics of *C. auris*?

*C. auris* was first described after it was isolated from the ear canal of a 70-year-old Japanese woman at Tokyo Metropolitan Geriatric Hospital in Japan (Satoh et al., 2009). After this report, a retrospective review of *Candida* strain collections revealed that the earliest known strain of *C. auris* dates back to 1996 in South Korea (Kim et al., 2009). However, it is unknown why *C. auris* has only recently emerged in a wide variety of locations. Based on whole genome sequencing (WGS), four different clades of *C. auris* have been described by region (East Asian, South Asian, African, and South American). Moreover, analyses of SNPs identified by WGS and multilocus sequence typing (MLST) have shown low genetic diversity between isolates within each *C. auris* clade. These results indicate a high degree of clonality within clades and suggest the independent, nearly simultaneous emergence of the four populations on three continents (Lockhart et al., 2017).

The complete genome of *C. auris* was only recently investigated (Chatterjee et al., 2015; Sharma et al., 2015, 2016), and we are far from fully understanding of the role of different genes in the pathogenicity and virulence of this emerging pathogen. The main problem is that the *C. auris* genome sequence contains many uncharacterized and hypothetical proteins, and it is unclear whether these proteins are involved in species-specific characteristics that promote its aggressiveness as a pathogen (Chatterjee et al., 2015). *C. auris* is closely related to three other rare *Candida* species – *Candida haemulonii*, *Candida duobushaemulonii*, and *Candida pseudohaemulonii* – in the family Metschnikowiaceae, along with more common *Candida lusitaniae* (Cendejas-Bueno et al., 2012).

Initial analyses of the *C. auris* genome suggest that 40% of *C. auris* proteins are orthologous to those of *C. lusitaniae* (Sharma et al., 2016). Considering the limited susceptibility of *C. lusitaniae* to amphotericin B and *C. haemulonii* to amphotericin B and azoles (Kim et al., 2009; Espinel-Ingroff et al., 2017), this orthology suggests that *C. auris* shares some genetic characteristics with both species that may have helped it become resistant to multiple antifungal drugs. Indeed, a significant portion of the *C. auris* genome encodes transporter genes and protein kinases, such as the ABC and MFS transporter families, and a number of zinc cluster transcription factor orthologs such as *TAC1* (29% similarity with *Candida albicans*), which may facilitate acquisition of drug resistance (Sharma et al., 2016).

By comparing the *C. auris* genome with that of *C. albicans* that is well annotated and well-studied we may suggest that C. *auris* genome harbors genes that are well-characterized as virulence factors in other *Candida* species, including genes for biofilm formation, proteinases, lipases, phospholipases, adhesins, secreted aspartyl proteases and transporters belonging to the ABC and major facilitator superfamily (MDR transcription factors) which are involved with azole resistance (Chatterjee et al., 2015).

## Phenotypic Traits and Implications for Pathogenicity

Phenotypic switching and morphogenesis are well-known virulence attributes of *C. albicans* (Polke et al., 2015). Although micromorphological studies of *C. auris* colonies suggest that this pathogen does not produce germ tubes, pseudohyphae or chlamydospores (Lee et al., 2011; Chowdhary et al., 2014; Borman et al., 2016; Wang et al., 2018), this yeast may present pseudohyphae-like forms under high-salt stress (Wang et al., 2018) and, occasionally, in the biofilm community (Sherry et al., 2017).Pseudohyphae-like forms are characterized by rudimentary growth, with an elongated shape and incomplete cell division (Sherry et al., 2017; Wang et al., 2018). Indeed, comparative genome analyses have confirmed that *C. auris* and closely related species do not have two genes, candidalysin (*ECE1*) and hyphal cell wall protein (*HWP1*), both of which are highly expressed in *C. albicans* and the transcription of both is strongly associated with hyphal formation (Munoz et al., 2018).

In this scenario, *C. auris* fails to form chlamydospores after growth on cornmeal agar when incubated for 3 days at 30°C and does not germinate when incubated with fetal bovine serum (FBS). This fact was confirmed in the *Galleria mellonella* model of infection of *C. auris*, during which the isolates did not undergo significant filamentation at 18 h or at any time post infection (Borman et al., 2016).

Growth curve analyses for *C. auris* and *C. albicans* isolates demonstrate that the two *Candida* species have similar growth patterns, reaching the stationary phase within approximately 20 h (Larkin et al., 2017). An interesting observation regarding the growth of *C. auris* is that certain isolates grow in clumps (i.e., budding occurs, but daughter cells are not released), which results in large aggregates of organisms that cannot be easily disrupted *in vitro* (Borman et al., 2016). These isolates are called “aggregate” strains, and certain aggregate strains are too large for *G. mellonella* larval inoculation, hindering accurate yeast cell counting. To overcome this limitation, homogeneous inoculum preparations have been obtained after allowing the initial suspension of fungal cells to settle for 10 min followed by removal of the supernatant containing individual yeast cells for infecting larvae (Borman et al., 2016). Based on this protocol, it was possible to compare the pathogenicity of aggregating and non-aggregating strains of *C. auris* with other yeast species. The results demonstrated strain-specific variability in behavior, with the aggregate-forming isolates of *C. auris* exhibiting significantly reduced pathogenicity compared to their non-aggregating counterparts (Borman et al., 2016). Moreover, when infected *G. mellonella* larvae were dissected, large numbers of individual budding yeast cells of non-aggregating strains of *C. auris* were found inside phagocytes. In addition, the larvae inoculated with individual yeast cells prepared from aggregate-forming strains of *C. auris* exhibited large aggregates of *C. auris* cells, with few individual yeast cells, indicating that the ability to produce large aggregates had been maintained *in vivo* (Borman et al., 2016). Yeast cell aggregates were also observed in the kidneys of mice infected with *C. auris,* suggesting that aggregation might be a mode of immune evasion and persistence in tissues, which warrants further investigation (Ben-Ami et al., 2017).

## Tolerance to Thermic and Osmotic Stresses

Survival and growth at physiologic temperature are prerequisites for microbial invasion and pathogenicity. *C. auris* exhibits thermotolerance, growing optimally at 37°C and maintaining viability up to 42°C. In addition, this pathogen is salt tolerant, and cells aggregate into large, difficult-to-disperse clusters, which may promote persistence in the hospital environment (Satoh et al., 2009; Borman et al., 2016).

The ability of *C. auris* isolates to grow at 37°C and 40°C appears to be similar to that of *C. albicans*, and certain isolates also grow at 42°C (Ben-Ami et al., 2017). Other authors have confirmed that *C. auris* can grow at high temperature (40°C) and salinity (10%wt/vol) when cultured in Sabouraud (SAB) or yeast nitrogen base (YNB) broth with dulcitol or mannitol as the carbon source (Welsh et al., 2017).

Wang et al. (2018) examined the morphology of *C*. *auris* on the rich media YPD and YPD plus 10% NaCl, with a round appearance on the former medium but an elongated shape on the latter. Interestingly, a small portion of highly elongated and pseudohyphae-like forms were observed when grown on YPD plus 10% NaCl (Wang et al., 2018). These results suggest that high-salt stress may result in incomplete cell division, leading to the formation of pseudohyphae-like forms. So far, the molecular mechanism related to this phenotype of *C. auris* has not yet been investigated.

## Importance of Lytic Enzymes for Fungal Invasiveness

The production of phospholipases and proteinases has been recognized as relevant for *Candida* pathogenicity in the human host, helping in the adherence to and invasion of host cells (Polke et al., 2015). Hydrolases are the largest group of enzymes (42%) found in the *C. auris* (strain 6684) genome, followed by transferases (25%) and oxidoreductases (19%) (Chatterjee et al., 2015). Furthermore, comparative genome analyses have revealed similar numbers of lipases in the *C. auris* relative to those of C. *albicans* and *C*. *dubliniensis* (Munoz et al., 2018). The ability to produce lytic enzymes has been demonstrated in *C. auris* isolates, and the production of these enzymes is strain dependent (Kumar et al., 2015; Larkin et al., 2017).

An *in vitro* study evaluating *C. auris* isolates from different geographical regions showed that 37.5% of the tested strains (6/16 isolates) exhibited phospholipase activity and that 64% (9/14 isolates) were positive for proteinase (Larkin et al., 2017). Similarly, most strains displayed hemolysin activity, conferring a high capacity for iron acquisition, growth, and invasiveness leading to widespread infection (Tsang et al., 2007; Kumar et al., 2015).

In fact, a recent study conducted by Wang et al. (2018) demonstrated that the level of aspartyl proteinase (Saps) secreted by *C. auris* isolate at 42°C was higher than that exhibited by *C. albicans* at the same temperature (Wang et al., 2018). These findings suggest that *C. auris* isolates are not only well adapted to temperature stress but also maintain their pathogenicity at higher temperatures.

## *C. auris* and Its Alarming Ability to Persistently Colonize Human Host and the Environment

Studies have demonstrated the ability of *C. auris* to colonize and spread throughout hospital environments. One of the most alarming characteristics of *C. auris* is the ability of this yeast to adhere to and persist on abiotic surfaces, including dry and moist surfaces, bedding material, floors, sinks and beds, as well as human skin, ears, and nasal cavities (Schelenz et al., 2016; Piedrahita et al., 2017; Vallabhaneni et al., 2017; Welsh et al., 2017). In one study, the survival of eight *C. auris* isolates was compared with other *Candida* species on dry or moist surfaces for up to 7 days, with the recovery of *C. auris* being similar to that of other *Candida* species, vancomycin-resistant *Enterococcus* (VRE), methicillin-resistant *Staphylococcus aureus* (MRSA), and carbapenem-resistant Enterobacteriaceae (CRE) (Piedrahita et al., 2017).

Moreover, the capacity of *C. auris* to persist and colonize the plastic surfaces of medical devices was compared with that of *Candida parapsilosis* (Welsh et al., 2017) using two complementary independent methods: culture [as measured by colony-forming units (CFU)] and solid-phase cytometry (which detects viability, as measured by esterase activity) (Smith et al., 2010). For CFU determination, *C. auris* and *C. parapsilosis* suspensions of 5 × 10^6^ cells per mL were applied to dried plastic surfaces and followed to grow for up to 28 days. *C. auris* and *C. parapsilosis* were viable for at least 14 and 28 days, respectively; however, viable *C. auris* was detected for longer periods than previously observed by quantitative culturing. When solid-phase cytometry was used to assess individual cells for viability for longer periods of time, the results indicated that the cells entered into a viable non-culturable state, and more viable *C. auris* than *C. parapsilosis* cells were detected at all time points (Welsh et al., 2017).Overall, the adherence of *C. auris* to medical devices may play a role in the development of catheter-related fungemia. In contrast to *C. albicans, C. auris* exhibits minimal ability to adhere to silicone elastomer (Larkin et al., 2017).

## Biofilm Production and Impact on Antifungal Resistance

Biofilms are a common form of microbial growth that are crucial for the development of a broad spectrum of infections in the human host and also for defending pathogens from phagocytes and antimicrobial drugs (Fanning and Mitchell, 2012). Seven highly conserved genes (*PLB3, IFF4, PGA52, PGA26, CSA1, HYR3,* and *PGA7*) are upregulated during biofilm production across isolates representative of *C. auris*, *C. haemulonii*, *C. duobushaemulonii*, and *C. pseudohaemulonii*. The same proteins are associated with biofilm production and mechanisms of antifungal resistance in *C. albicans* strains (Kean et al., 2018). As documented with other *Candida* species (Hirakawa et al., 2015), there is a large intra-specific variation in the capacity of biofilm production by *C. auris.* Initial investigation with fifteen *C. auris* isolates cultured from ear specimens failed to detect any biofilm production (Oh et al., 2011). Later, Larkin et al. (2017) performed a comparative study to assess biofilm production by two isolates of *C. auris* and one isolate of *C. albicans* using silicone elastomer as a substrate. Quantification of the biofilms was performed using a colorimetric metabolic assay (to measure mitochondrial dehydrogenase activity-XTT) and dry weight analysis (to measure total biofilm mass). During this assay, the authors were able to detect *C. auris* biofilm production that was composed of yeast cells adhering to the catheter material. In contrast, the *C. albicans* biofilm presented a highly heterogeneous architecture composed of yeast cells and hyphae embedded within the extracellular matrix. Moreover, the *C. auris* biofilms, unlike the *C. albicans* biofilms, displayed a limited amount of extracellular matrix (Larkin et al., 2017).

Recently, a comparative study was performed to evaluate the production of biofilm by isolates of *C. albicans, Candida glabrata,* two non-aggregating strains of *C. auris* and two aggregating strains of *C. auris.* The authors used 96-well polystyrene microtiter plates and measured total fungal biomass using a crystal violet assay. The results showed the greatest biofilm mass for *C. albicans*, followed by *C. auris* and *C. glabrata.* The *C. auris* biofilm was predominately composed of budding yeasts and occasional pseudohyphae (Sherry et al., 2017). In the same study, the susceptibility of *C. auris* biofilms to drugs was assessed, with micafungin and caspofungin being ineffective against biofilms “*in vitro*” and requiring > 32 mg/L to inhibit sessile cells (Sherry et al., 2017).

## Virulence of *C. auris* in Animal Models

A comparative study of virulence exhibited by *C. auris* and *C. haemulonii* isolates was performed in a mouse model of hematogenous-disseminated candidiasis after immunosuppression with cyclophosphamide (150 mg/kg intraperitoneally). The *C. haemulonii* isolates were found to be completely non-virulent, with 100% of the mice surviving at 12 days after inoculation and no visible signs of illness. In contrast, inoculation with *C. auris* resulted in rapid death, with only 20% survival at 5 days after infection (Munoz et al., 2018). Another comparative study of *Candida* spp. virulence using an immunocompetent murine model of disseminated infection showed high virulence of *C. albicans* isolates, followed by *C. auris*, *C. glabrata,* and *C. haemulonii* (Fakhim et al., 2018). In this study, the animals were challenged with 10^5^ CFU/mouse injected into the lateral tail vein, and death rates were recorded up to 30 days post-infection. Mice infected with *C. albicans* exhibited 20% survival, with a median survival time (MST) of 13 days, where as mice infected with *C. auris* showed 30–40% survival until the end of the experiment, with an MST of 16–17 days post-infection (Fakhim et al., 2018).

The virulence of a single *C*. *auris* isolate obtained from a Chinese fungemic patient was recently evaluated in a mouse model of *Candida* systemic infection. Inoculum preparations of 1 × 10^6^CFU/ml of *C*. *albicans* (SC5314) and 1 × 10^7^CFU/ml *C. auris* were injected via the tail vein, with all animals infected with *C. albicans* dying by the 6th day post-infection but no animal infected with *C. auris* dying during the same period (Wang et al., 2018).

Due to economic and especially ethical issues, the scientific community has in the last decade limited the use of mammalian infection models to study the virulome of fungi (Krappmann, 2015). Driven by the need for validating alternative models to replace mammalian systems, invertebrate organisms, such as *G. mellonella* and *C. elegans*, have been extensively used as mini-hosts for studying the virulence attributes and host response of *Candida* infections (Cotter et al., 2000).

Within this context, Borman et al. (2016) developed a model of *G. mellonella* fungal infection using an inoculum solution of 1 × 10^6^ yeast cells of *C. auris, C. albicans* and *Candida* *tropicalis* isolates, whereby it was possible to demonstrate that *C. albicans* and *C. tropicalis* exhibit significantly higher virulence in terms of the kinetics of larval death and number of larvae killed (Borman et al., 2016). In accordance with other publications, these researchers found that *C. auris* isolates were not able to develop significant pseudohyphae into tissues of infected larvae (Lee et al., 2011; Chowdhary et al., 2013; Kathuria et al., 2015; Borman et al., 2016). Wang et al. (2018) also confirmed that the Chinese *C. auris* strain exhibits reduced virulence in *G*. *mellonella* larva compared to a *C. albicans* isolate. Overall, compared to *C. glabrata*, *C. auris* strains are usually more virulent in animal models.

Investigation of the immune response to *C. auris* using the Zebrafish model of invasive candidiasis revealed a recruitment of approximately 50% less neutrophils in response to *C. auris* infection when compared to *C. albicans* (Johnson et al., 2018). Moreover, *in vitro* human neutrophils were co-cultured with *Candida* cells (2 × 10^7^ cells) for 4 h and fungal viability was measured. Neutrophils inhibited *C. albicans* growth by 75%; in contrast, the burden of *C. auris* was not impacted replicating beyond the initial inoculums, showing very little killing of *C. auris* by neutrophils. Using fluorescence microscopy, they analyzed neutrophil-*Candida* interactions, and at 1 h, very few neutrophils (15%) were either engulfing or adherent to co-incubated *C. auris* cells. In contrast, 50% of neutrophils exhibited some activity against *C. albicans.* These findings suggest that neutrophils are more able to engage and kill *C. albicans* over *C. auris* isolates (Johnson et al., 2018). Viability of neutrophils co-cultured with *C. albicans* decreased by half, while upon exposure to *C. auris,* almost all neutrophils remained viable. The production of neutrophils extracellular traps (NETs) in response to *C. auris* exposition was measured by scanning electron microscopy. It was found that neutrophils engaging in phagocytosis or releasing NETs were rarely observed after exposition to *C. auris*. Furthermore, quantifying NET formation showed that *C. auris* did not trigger free DNA release, consistent with the lack of NET production observed in microscopy experiments (Johnson et al., 2018).

Taking together data provided by experimental models using mice and invertebrate animals, we may conclude that *C. auris* is certainly less virulent than is *C. albicans*. Nonetheless, *C. auris* is significantly more virulent than are *C. glabrata* and *C. haemulonii* (Fakhim et al., 2018) and other species of *Candida* also considered to be resistant to multiple drugs. Indeed, *C. auris* was able to induce systemic infection and mortality rates higher than 50% in 2 of 3 studies investigating its virulence in a mouse model (Ben-Ami et al., 2017; Fakhim et al., 2018; Wang et al., 2018). Despite limited virulence when compared to *C. albicans*, *in vitro* assays conducted with human neutrophils showed that *C. auris* is less effective than *C. albicans* in triggering neutrophils engulfment and NET production.

## Final Comments and Future Directions

Although animal studies indicate that *C. auris* has reduced pathogenicity and virulence compared to *C. albicans*, this emerging pathogen appears to be far more able to induce systemic infection and mortality than other potential MDR yeast pathogens, such as *C. glabrata* and *C. haemulonii* (Fakhim et al., 2018). This finding is likely to be related to the tolerance of *C. auris* strains to osmotic and high-temperature stress as well as to its ability to produce several lytic enzymes and biofilm (Ben-Ami et al., 2017; Welsh et al., 2017).

The complete genome sequence of *C. auris* is available (Chatterjee et al., 2015; Sharma et al., 2015; Lockhart et al., 2017), and it is currently possible to design experiments to better characterize the molecular mechanisms responsible for the capability of this pathogen to readily become resistant to multiple antifungal drugs and to determine the presence of genes related to pathogenicity and virulence factors. An important limitation of virulence analyses based on clonal strains cultured from patients during outbreaks is that it remains unclear whether such findings may be safely extrapolated to all isolates of the species or whether they are only representative of biological properties exhibited by a few clonal strains.

An additional point that deserves more attention is the characterization of adhesins and other molecules responsible for the capability of *C. auris* to persistently colonize abiotic and biotic surfaces. Indeed, this pathogen is able to survive and persist under different environmental conditions, including on dry materials, bedding material, floors, sinks and beds, and it exhibits tolerance to temperature and osmotic stresses (Ben-Ami et al., 2017; Welsh et al., 2017).

In summary, our review shows that *C. auris* expresses several virulence traits including genes that are well-characterized in other *Candida* species, including genes for biofilm formation, proteinases, lipases, phospholipases, adhesins, secreted aspartyl proteases, and transporters which are involved with azole resistance. Despite exhibiting less virulence than *C. albicans* isolates, recent findings suggest that *C. auris* fails to activate the innate immune response and production of NETs by human neutrophils, what certainly may play a role in the high mortality associated to this infection.

## Author Contributions

LR and AC contributed equally in the paper. Both wrote the manuscript and performed all the necessary literature searches and data compilation.

## Conflict of Interest Statement

AC received educational grants from Pfizer, Gilead Sciences – United Medical (Brazil), MSD, and research grants from Astellas and Pfizer. The remaining author declares that the research was conducted in the absence of any commercial or financial relationships that could be construed as a potential conflict of interest.

## References

[B1] Ben-Ami R. Berman J. Novikov A. Bash E. Shachor-Meyouhas Y. Zakin S. (2017). Multidrug-Resistant Candida haemulonii and C. auris, Tel Aviv, Israel. *Emerging Infectious Diseases.* 23 195–203. 10.3201/eid2302.161486 28098529 PMC5324804

[B2] Borman A. M. Szekely A. Johnson E. M. (2016). Comparative Pathogenicity of United Kingdom Isolates of the Emerging Pathogen Candida auris and Other Key Pathogenic Candida Species. *mSphere.* 1 e189–e116. 10.1128/mSphere.00189-16 27547827 PMC4990711

[B3] Calvo B. Melo A. S. Perozo-Mena A. Hernandez M. Francisco E. C. Hagen F. (2016). First report of Candida auris in America: Clinical and microbiological aspects of 18 episodes of candidemia. *J Infect.* 73 369–374. 10.1016/j.jinf.2016.07.008 27452195

[B4] Cendejas-Bueno E. Kolecka A. Alastruey-Izquierdo A. Theelen B. Groenewald M. Kostrzewa M. (2012). Reclassification of the Candida haemulonii Complex as Candida haemulonii (C. *haemulonii Group* I), C. duobushaemulonii sp. nov. (C. haemulonii Group II), and C. haemulonii var. vulnera var. nov.: Three Multiresistant Human Pathogenic Yeasts. *Journal of Clinical Microbiology.* 50 3641–3651. 10.1128/JCM.02248-12 22952266 PMC3486233

[B5] Chatterjee S. Alampalli S. V. Nageshan R. K. Chettiar S. T. Joshi S. Tatu U. S. (2015). Draft genome of a commonly misdiagnosed multidrug resistant pathogen Candida auris. *BMC Genomics.* 16:686. 10.1186/s12864-015-1863-z 26346253 PMC4562351

[B6] Chowdhary A. Anil Kumar V. Sharma C. Prakash A. Agarwal K. Babu R. (2014). Multidrug-resistant endemic clonal strain of Candida auris in India. *Eur J Clin Microbiol Infect Dis.* 33 919–926. 10.1007/s10096-013-2027-1 24357342

[B7] Chowdhary A. Sharma C. Duggal S. Agarwal K. Prakash A. Singh P. K. (2013). New Clonal Strain of Candida auris, Delhi, India: New Clonal Strain of Candida auris, Delhi, India. *Emerging Infectious Diseases.* 19 1670–1673. 10.3201/eid1910.130393 24048006 PMC3810747

[B8] Cotter G. Doyle S. Kavanagh K. (2000). Development of an insect model for the in vivo pathogenicity testing of yeasts. *FEMS Immunol Med Microbiol.* 27 163–169. 10.1111/j.1574-695X.2000.tb01427.x 10640612

[B9] Espinel-Ingroff A. Arendrup M. Cantón E. Cordoba S. Dannaoui E. García-Rodríguez J. (2017). Multicenter Study of Method-Dependent Epidemiological Cutoff Values for Detection of Resistance in Candida spp. *and Aspergillus spp. to Amphotericin B and Echinocandins for the Etest Agar Diffusion Method*. *Antimicrobial Agents and Chemotherapy.* 61 e1792–e1716. 10.1128/AAC.01792-16 27799206 PMC5192158

[B10] Fakhim H. Vaezi A. Dannaoui E. Chowdhary A. Nasiry D. Faeli L. (2018). Comparative virulence of Candida auris with Candida haemulonii, Candida glabrata and Candida albicans in a murine model. *Mycoses* 61 377–382. 10.1111/myc.12754 29460345

[B11] Fanning S. Mitchell A. P. (2012). Fungal Biofilms. *PLoS Pathogens.* 8:e1002585. 10.1371/journal.ppat.1002585 22496639 PMC3320593

[B12] Hirakawa M. P. Martinez D. A. Sakthikumar S. Anderson M. Z. Berlin A. Gujja S. (2015). Genetic and phenotypic intra-species variation in Candida albicans. *Genome research.* 25 413–425. 10.1101/gr.174623.114 25504520 PMC4352881

[B13] Johnson C. J. Davis J. M. Huttenlocher A. Kernien J. F. Nett J. E. (2018). Emerging Fungal Pathogen Candida auris Evades Neutrophil Attack. *MBio.* 9 e01403. 10.1128/mBio.01403-18 30131360 PMC6106086

[B14] Kathuria S. Singh P. K. Sharma C. Prakash A. Masih A. Kumar A. (2015). Multidrug-Resistant Candida auris Misidentified as Candida haemulonii: Characterization by Matrix-Assisted Laser Desorption Ionization–Time of Flight Mass Spectrometry and DNA Sequencing and Its Antifungal Susceptibility Profile Variability by Vitek 2, CLSI Broth Microdilution, and Etest Method. *Journal of Clinical Microbiology.* 53 1823–1830. 10.1128/JCM.00367-15 25809970 PMC4432077

[B15] Kean R. Delaney C. Sherry L. Borman A. Johnson E. M. Richardson M. D. (2018). Transcriptome Assembly and Profiling of Candida auris Reveals Novel Insights into Biofilm-Mediated Resistance. *mSphere* 3 e334–e318. 10.1128/mSphere.00334-18 29997121 PMC6041501

[B16] Kim M. N. Shin J. H. Sung H. Lee K. Kim E. C. Ryoo N. (2009). Candida haemulonii and closely related species at 5 university hospitals in Korea: identification, antifungal susceptibility, and clinical features. *Clin Infect Dis.* 48 e57–e61. 10.1086/597108 19193113

[B17] Krappmann S. (2015). Lightning up the worm: How to probe fungal virulence in an alternative mini-host by bioluminescence. *Virulence* 6 727–729. 10.1080/21505594.2015.1103428 26537579 PMC4826133

[B18] Kumar D. Banerjee T. Pratap C. B. Tilak R. (2015). Itraconazole-resistant Candida auris with phospholipase, proteinase and hemolysin activity from a case of vulvovaginitis. *Journal of infection in developing countries.* 9 435–437. 10.3855/jidc.4582 25881537

[B19] Larkin E. Hager C. Chandra J. Mukherjee P. K. Retuerto M. Salem I. (2017). The emerging pathogen candida auris: growth phenotype, virulence factors, activity of antifungals, and effect of SCY-078, a novel glucan synthesis inhibitor, on growth morphology and biofilm formation^∗^. *Antimicrob Agents Chemother.* 61 10.1128/aac.02396-16 28223375 PMC5404565

[B20] Lee W. G. Shin J. H. Uh Y. Kang M. G. Kim S. H. Park K. H. (2011). First three reported cases of nosocomial fungemia caused by Candida auris. *J Clin Microbiol.* 49 3139–3142. 10.1128/jcm.00319-11 21715586 PMC3165631

[B21] Lockhart S. R. Etienne K. A. Vallabhaneni S. Farooqi J. Chowdhary A. Govender N. P. (2017). Simultaneous Emergence of Multidrug-Resistant Candida auris on 3 Continents Confirmed by Whole-Genome Sequencing and Epidemiological Analyses. *Clin Infect Dis.* 64 134–140. 10.1093/cid/ciw691 27988485 PMC5215215

[B22] Morales-Lopez S. E. Parra-Giraldo C. M. Ceballos-Garzon A. Martinez H. P. Rodriguez G. J. Alvarez-Moreno C. A. (2017). Invasive Infections with Multidrug-Resistant Yeast Candida auris, Colombia. *Emerg Infect Dis.* 23 162–164. 10.3201/eid2301.161497 27983941 PMC5176232

[B23] Munoz J. F. Gade L. Chow N. A. Loparev V. N. Juieng P. Farrer R. A. (2018). Genomic basis of multidrug-resistance, mating, and virulence in Candida auris and related emerging species. *bioRxiv* [Preprint] 10.1101/299917 PMC6297351 30559369

[B24] Oh B. J. Shin J. H. Kim M. N. Sung H. Lee K. Joo M. Y. (2011). Biofilm formation and genotyping of Candida haemulonii, Candida pseudohaemulonii, and a proposed new species (Candida auris) isolates from Korea. *Med Mycol.* 49 98–102. 10.3109/13693786.2010.493563 20560864

[B25] Piedrahita C. T. Cadnum J. L. Jencson A. L. Shaikh A. A. Ghannoum M. A. Donskey C. J. (2017). Environmental Surfaces in Healthcare Facilities are a Potential Source for Transmission of Candida auris and Other Candida Species. *Infection control and hosp. epidemiology.* 38 1107–1109. 10.1017/ice.2017.127 28693657

[B26] Polke M. Hube B. Jacobsen I. D. (2015). Candida survival strategies. *Advances in Applied Microbiology.* 91 139–235. 10.1016/bs.aambs.2014.12.002 25911234

[B27] Prakash A. Sharma C. Singh A. Kumar Singh P. Kumar A. Hagen F. (2016). Evidence of genotypic diversity among Candida auris isolates by multilocus sequence typing, matrix-assisted laser desorption ionization time-of-flight mass spectrometry and amplified fragment length polymorphism. *Clin Microbiol Infect.* 22 277.e1–277.e9. 10.1016/j.cmi.2015.10.022 26548511

[B28] Sarma S. Kumar N. Sharma S. Govil D. Ali T. Mehta Y. (2013). Candidemia caused by amphotericin B and fluconazole resistant Candida auris. *Indian J Med Microbiol.* 31 90–91. 10.4103/0255-0857.108746 23508441

[B29] Satoh K. Makimura K. Hasumi Y. Nishiyama Y. Uchida K. Yamaguchi H. (2009). Candida auris sp. *nov., a novel ascomycetous yeast isolated from the external ear canal of an inpatient in a Japanese hospital*. *Microbiology and immunology.* 53 41–44. 10.1111/j.1348-0421.2008.00083.x 19161556

[B30] Schelenz S. Hagen F. Rhodes J. L. Abdolrasouli A. Chowdhary A. Hall A. (2016). First hospital outbreak of the globally emerging Candida auris in a European hospital. *Antimicrob Resist Infect Control.* 5 35. 10.1186/s13756-016-0132-5 27777756 PMC5069812

[B31] Sharma C. Kumar N. Meis J. F. Pandey R. Chowdhary A. (2015). Draft Genome Sequence of a Fluconazole-Resistant Candida auris Strain from a Candidemia Patient in India. *Genome Announc.* 3 e722–e715. 10.1128/genomeA.00722-15 26184929 PMC4505117

[B32] Sharma C. Kumar N. Pandey R. Meis J. F. Chowdhary A. (2016). Whole genome sequencing of emerging multidrug resistant Candida auris isolates in India demonstrates low genetic variation. *New Microbes and New Infections.* 13 77–82. 10.1016/j.nmni.2016.07.003 27617098 PMC5006800

[B33] Sherry L. Ramage G. Kean R. Borman A. Johnson E. M. Richardson M. D. (2017). Biofilm-Forming Capability of Highly Virulent, Multidrug-Resistant Candida auris. *Emerg Infect Dis.* 23 328–331. 10.3201/eid2302.161320 28098553 PMC5324806

[B34] Smith R. Von Tress M. Tubb C. Vanhaecke E. (2010). Evaluation of the ScanRDI(R) as a Rapid Alternative to the Pharmacopoeial Sterility Test Method: Comparison of the Limits of Detection. *PDA J. Pharm. Sci. Technol.* 64 356–363. 21502036

[B35] Tsang C. S. Chu F. C. Leung W. K. Jin L. J. Samaranayake L. P. Siu S. C. (2007). Phospholipase, proteinase and haemolytic activities of Candida albicans isolated from oral cavities of patients with type 2 diabetes mellitus. *J Med Microbiol.* 56 (Pt 10), 1393–1398. 10.1099/jmm.0.47303-0 17893179

[B36] Vallabhaneni S. Kallen A. Tsay S. Chow N. Welsh R. Kerins J. (2017). Investigation of the First Seven Reported Cases of Candida auris, a Globally Emerging Invasive, Multidrug-Resistant Fungus-United States, May 2013-August 2016. *American Journal of Transplantation* 17 296–299. 10.1111/ajt.14121 28029734

[B37] Wang X. Bing J. Zheng Q. Zhang F. Liu J. Yue H. (2018). The first isolate of Candida auris in China: clinical and biological aspects. *Emerging Microbes & Infections.* 7 93. 10.1038/s41426-018-0095-0 29777096 PMC5959928

[B38] Welsh R. M. Bentz M. L. Shams A. Houston H. Lyons A. Rose L. J. (2017). Survival, Persistence, and Isolation of the Emerging Multidrug-Resistant Pathogenic Yeast Candida auris on a Plastic Health Care Surface. *J Clin Microbiol.* 55 2996–3005. 10.1128/jcm.00921-17 28747370 PMC5625385

